# Editorial: Inflammasomes in infectious diseases, cell death and ROS generation: stimulation by microorganisms and membrane-derived microvesicles

**DOI:** 10.3389/fimmu.2024.1378506

**Published:** 2024-02-27

**Authors:** Prescilla Emy Nagao, Louisy Santos, A. Marijke Keestra-Gounder

**Affiliations:** ^1^ Laboratory of Molecular Biology and Physiology of Streptococci, Institute of Biology Roberto Alcantara Gomes, Rio de Janeiro State University – UERJ, Rio de Janeiro, Brazil; ^2^ Laboratory of Diphtheria and Corynebacteria of Clinical Relevance, Faculty of Medical Sciences, Rio de Janeiro State University – UERJ, Rio de Janeiro, Brazil; ^3^ Department of Immunology and Microbiology, University of Colorado Anschutz Medical Campus, Aurora, CO, United States

**Keywords:** inflammasome, infectious diseases, pyroptosis and apoptosis, extracellular vesicles, reactive oxygen species

Microbial infections and damage to host cells can activate inflammasomes, which mediate tissue inflammation and adaptive immunity. Inflammasomes are innate immune mechanisms that promote inflammation by activating caspase-1 protease and are associated with cell death, such as pyroptosis and apoptosis. Although inflammasome and IL-1β signaling are crucial in tissue inflammation, little has been known about their regulatory mechanism. Inflammasome activation and subsequent pyroptosis are critical defense mechanisms against pathogenic microorganisms. However, overactivation of the inflammasome leads to the death of the host (1, 2). Activation of the inflammasome by Gram-negative bacteria or non-canonical activation of the inflammasome by lipopolysaccharide (LPS) can also induce systemic blood clotting and massive tissue thrombosis (3).

Inflammasome-induced extracellular vesicles (EVs) can serve as biomarkers for inflammasome activation and act in the prevention of systemic hyperinflammatory states by restricting NLRP3 activation in eukaryotic cells (4). The NLRP3 inflammasome is activated by diverse stimuli and multiple molecular and cellular events, including infections by microorganisms, production of reactive oxygen species, and lysosomal damage. Inflammasomes can cause mitochondrial fission with the release of microvesicles into host cells, resulting in disordered function in capillaries and venules (5, 6). Understanding the molecular mechanisms of inflammasome-associated cell death and its physiological implications may contribute to the development of new therapeutic strategies for inflammasome-related infectious diseases.

We thank all the authors who provided relevant aspects of inflammasomes and infectious diseases to this Research Topic by analyzing different features concerning virulence, innate immune responses, potential molecular targets, diagnostic application, and biomarkers.

*Streptococcus equi* subsp. *zooepidemicus* (SEZ) is an essential zoonotic bacterial pathogen that can cause various inflammation, such as meningitis, endocarditis, and pneumonia. The study conducted by Xu et al. showed that gasdermin D (GSDMD) is involved in pore formation, cytokine release and cell death, indicating an important role in controlling the microbial infection. These findings highlight the host defense mechanisms of GSDMD against SEZ infection, providing a potential therapeutic target in SEZ infection.

Previous reviews have emphasized the conservation of inflammasome components in evolution, including the mechanism that induces pyroptosis in fish. The review article written by Chang summarized the mechanisms of activation of canonical and non-canonical inflammasomes in teleost fish, with a particular focus on inflammasome complexes in response to bacterial infection. Greater knowledge of inflammasome activation and pathogen clearance in teleost fish will shed new light on molecular targets for treatment of inflammatory and infectious diseases.

The inflammasomes are intracellular multimeric protein complexes consisting of an innate immune sensor, the adapter protein ASC and the inflammatory caspases-1 and/or -11 that are important for the host defense against pathogens. Inflammasomes can detect a range of microbial ligands through direct or indirect interaction, including changes in ion fluxes, production of reactive oxygen species and disruption of various host cell functions. In their review, Keestra-Gounder and Nagao focused on the NLRP3, NLRP6, NLRC4 and AIM2 inflammasomes and how they are activated and regulated during infections with Gram-positive bacteria, including *Staphylococcus* spp., *Streptococcus* spp. and *Listeria monocytogenes*.

Tuberculosis is a major infectious disease induced by *Mycobacterium tuberculosis* (M. tb) which causes the world’s dominant fatal bacterial contagious disease. Increasing studies have indicated that exosomes may be a novel option for the diagnosis and treatment of tuberculosis. Exosomes are nanovesicles containing lipids, proteins and non-coding RNAs (ncRNAs) released from various cells, and can transfer their cargos and communicate between cells. Furthermore, exosomal ncRNAs exhibit diagnosis potential in bacterial infections, including tuberculosis. Additionally, differential exosomal ncRNAs regulate the physiological and pathological functions of M. tb-infected cells and act as diagnostic markers for tuberculosis. In their review, Wang et al. explored the potential biological roles and the diagnostic application prospects of exosomal ncRNAs, and included recent information on their pathogenic and therapeutic functions in tuberculosis.

The Notch signaling pathway has also been implicated in the pathogenesis of active tuberculosis, and Th1-type cell-mediated immunity is essential for effective control of mycobacterial infection. Xie et al. demonstrated that mRNA expression levels of Notch1, DLL1, and Hes1 were upregulated in active tuberculosis patients, with higher levels observed in those with moderate/severe tuberculosis than those with mild tuberculosis or without tuberculosis. Notably, Notch pathway molecules were more effective than Th1-type factors and white blood cell parameters in differentiating mild and moderate/severe cases of active tuberculosis. The study demonstrated that Notch1, Hes1, IFN-γ, and lymphocytes % can be used as biomarkers to identify different stages of active tuberculosis patients and to monitor the effectiveness of treatment.

The causal relationship between the gut microbiota and sepsis-related death is still unclear. Shang et al. employed mendelian randomization (MR) to determine whether a causal link exists between the two. This study used publicly available genome-wide association studies summary data of gut microbiota, sepsis, sepsis (critical care), and sepsis (28-day death in critical care) to perform a two-sample MR analysis. To ensure the robustness of the results, the authors also conducted a sensitivity analysis. The MR study has identified several bacteria that were causally linked to the development and progression of sepsis, providing new ideas for early diagnosis, personalized treatment, and outcome prognosis of sepsis.

In conclusion, this Research Topic updates important aspects of inflammasome activation in infectious diseases, providing examples of progress made in the field of microbial immunology over the last decade. The role of inflammasome activation depends on the nature of its interactions with specific microorganisms, which can promote protection or damage to the host. Modulation of some of these interactions could represent new therapeutic targets to combat infectious diseases and new ideas for early diagnosis, treatment and prognosis of sepsis.

## Author contributions

PN: Writing – review & editing, Conceptualization, Methodology, Project administration; LS: Writing – original draft, Methodology; AMK-G: Writing – review & editing, Formal Analysis.
